# Effect of *In Situ* short–term temperature increase on carbon metabolism and dissolved organic carbon (DOC) fluxes in a community dominated by the seagrass *Cymodocea nodosa*

**DOI:** 10.1371/journal.pone.0210386

**Published:** 2019-01-14

**Authors:** Luis G. Egea, Rocío Jiménez–Ramos, Ignacio Hernández, Fernando G. Brun

**Affiliations:** Department of Biology, Faculty of Marine and Environmental Sciences, University of Cadiz, Puerto Real (Cádiz), Spain; Università della Calabria, ITALY

## Abstract

Seagrasses form one of the most productive and threatened ecosystems worldwide because of global change and anthropogenic pressures. The frequency of extreme climatic events, such as heat waves, are expected to increase and may drive even more adverse effects than gradual warming. This study explores for the first time the effects of a sudden and temporary increase of temperature *in situ* on carbon metabolism and dissolved organic carbon (DOC) fluxes in a community dominated by a seagrass (*Cymodocea nodosa*) during two contrasting seasons (winter and summer). Results showed a positive correlation between temperature and seagrass production between seasons, while the experimental sudden and temporary increase in water temperature did not produce significant differences in carbon community metabolism and DOC fluxes in winter. In contrast, high temperature conditions in summer enhanced significantly the net community production and affected positively to DOC fluxes. Hence, this study indicates that a sudden and temporary increase in water temperature, which characterize marine heat waves, in temperate areas may enhance the autotrophic metabolism of seagrass communities and can yield an increase in the DOC released, in contrast to previous researches suggesting solely negative effects on seagrasses.

## Introduction

Global warming is emerging as a major threat to ecosystems worldwide [1, 2]. Mean global sea–surface temperatures have increased by 0.8°C over the last century [3] and, by the end of this century, is projected to increase by 3–4°C [4]. Besides mean sea–surface temperature alteration as a consequence of global change, the frequency and magnitude of extreme climatic events such as sudden and temporary increase of temperature–which characterizes the heat waves–across the globe are expected [4–6] Climate change research is generally concerned with the variation in ecosystems structure and functions associated with gradually increasing mean temperatures [7]. However, extreme climatic events such as heat waves will also dictate the response of ecosystems to climate change [8, 9]. Heat waves are usually defined as a period of anomalous increase of temperature and humidity [10] that, according to the World Meteorological Organization (WMO), has a duration of at least 2–3 days with a discernible impact on human and natural systems [11]. During heat wave events, the increase in air temperature usually translates into an increase in 2–4°C of the sea surface temperature (e.g. see Marbà & Duarte 2010 [12]). Although heat waves are usually associated with summer periods in the northern hemisphere, temporary and abnormal temperature rises can occur during all year, even in winter according to data from National Oceanic and Atmospheric Administration (NOAA) [13]. Understanding how ecosystems respond to extreme climatic events is necessary to predict how ecosystems and biodiversity will respond to climate change [14, 15]. In particular, understanding the response of communities dominated by foundation plant species (i.e. seagrasses) to extreme climatic events is essential as this will largely shape the ecological response at an ecosystem scale [16].

Seagrasses are marine foundation species that form one of the richest and most important coastal habitats [17]. They are globally distributed and well recognised by the ecosystem services they provide, such as high rates of productivity, coastal nutrient cycling, and support to other ecosystems as a habitat and food source [18, 19]. The shallow distribution of seagrasses and its proximity to anthropogenic littoral impacts has led to widespread seagrass losses with a global decline of 7% yr^-1^ [20]. This currently regression may be exacerbated by global change [21], including extreme temperature events [22–24], which may drive more impacts than gradual warming [12]. Seagrass meadows rank among the most productive ecosystems on Earth [25], which largely contribute to carbon uptake in coastal areas, while this carbon can be stored, consumed, buried or exported to adjacent ecosystems in the way of particulate or dissolved forms [26, 27]. Dissolved organic carbon (DOC) export from coastal ecosystems has been recently highlighted [28, 29] since it is a crucial, but not entirely understood part of the global carbon cycle. DOC is one of the largest interchangeable organic carbon reserves in the marine environment, being a central factor in the global carbon cycle [30, 31]. The DOC usually acts as a quick transfer of carbon in the food web because it is easily assimilated by marine organisms and fully involved in the carbon exchange between communities [30, 32–34]. The global net DOC export from seagrass meadows calculated by Barrón et al. (2014) [35] represents 46% of the global net community production (NCP) of seagrass meadows calculated by Duarte et al. (2010) [36]. Previous studies have shown that the net DOC fluxes in seagrass communities are significantly correlated with water temperature [27, 35], although these studies are based on seasonal monitoring programs. However, the effects of a sudden increase in water temperature on DOC fluxes in coastal vegetated habitats are largely unknown.

The effect of warming on seagrasses has been widely studied [37–40], including those recent *in situ* works (e.g. [41]) where unexpected responses were recorded when compared to laboratory–based studies, as a consequence of the integration of the whole community (i.e. sediment, fauna, macroalgae, epiphytes, plankton, etc) in the experimental design. Temperature is a key factor for seagrass health, growth and community metabolic rates [42, 43], but little attention has been given to the effects of sudden marine heat waves on seagrass carbon metabolism *in situ* including the whole community. To date, most studies related to heat waves have been carried out in terrestrial ecosystems (e.g. [44, 45]), with some of them recording a reduction in productivity at the ecosystem level [46–48]. Marine ecosystems also exhibit extreme ecological responses to these events. For instance, studies on coral reefs [49, 50], rocky benthic communities [51] and seaweeds [15] have reported widespread mortality or reduction in individuals abundance following marine heat waves. In seagrasses, most of the knowledge regarding the effects of heat waves is based on monitoring programs, which correlated seagrass shoot mortality with previous marine heat wave events in summer [12, 22, 52, 53]. However, *in situ* heat wave experiments have not been carried out to date. Bearing this in mind, the present study aims to gain insights into how a temperate seagrass community is affected by a sudden and temporary increase of temperature by analysing changes at the community level in carbon metabolism and DOC fluxes. Manipulating water temperatures *in situ* is a logistic challenge, which has not yet been addressed in marine heat waves research, in part as a consequence of the technical difficulties of promoting an increase in temperature *in situ*. However, this is an important step for understanding how the whole communities respond to temperature stress under natural conditions. Therefore, an *in situ* manipulative experiment, where a sudden an temporary temperature increase, as a proxy of a heat wave event, was designed and replicated in two seasons (winter and summer) to examine both the response of a community dominated by *Cymodocea nodosa* to a simulated heat wave, and the likely differential response under contrasting seasonal conditions.

## Material and methods

### Study area

**Fig 1:**
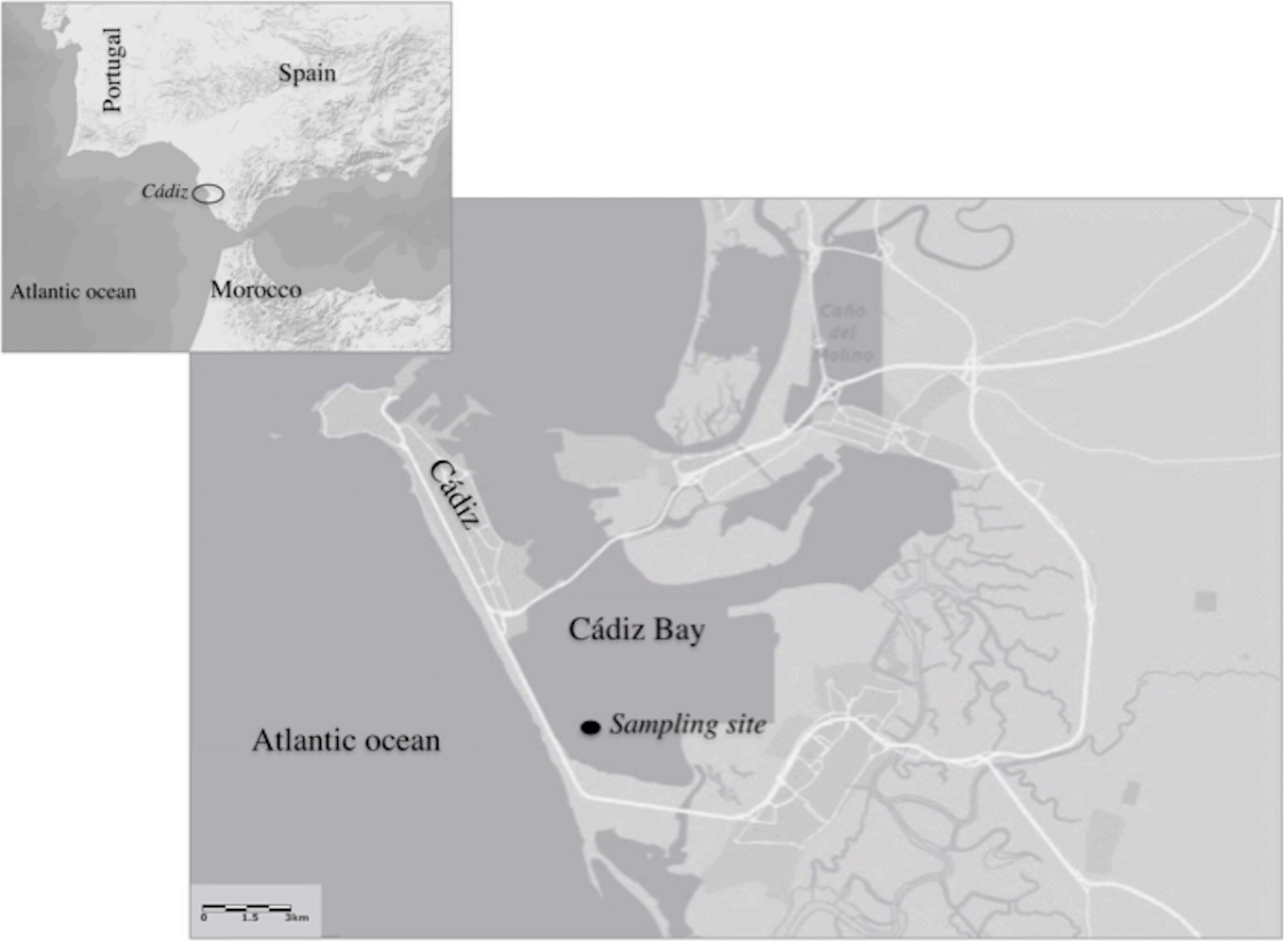
Study site at Cádiz bay (36° 28’ 12.79” N, 6° 15’ 7.07” W), spain.

This study was conducted in a subtidal community dominated by the seagrass *Cymodocea nodosa* growing at a depth of 3.5 m (low tide) in Santibañez, in the inner part of Cádiz bay, southern Spain (Fig 1). Climatically this area fits into a semi–warm subtropical thermal regime whose normal temperature range oscillates between 11 to 28°C and 593 mm as average annual precipitation. For detailed information of the study area, see previous descriptions in Morris et al. (2009) [54].

### Installation of incubations

The experiment was conducted in March (winter) and September (summer), from now called winter and summer trials respectively. Six areas, three replicates for control temperature (CT) and three replicates for high temperature (HT) were randomly selected within a large *C*. *nodosa* meadow, and benthic chambers (from now called incubations) were placed by scuba diving. The minimum distance between replicates was 6 m and the location of both CT and HT treatments were mixed in the meadow, avoiding any type of bias due to the location of the treatments (distance to coast, meadow density, etc). Although the community was distinctly dominated by *C*. *nodosa*, is actually an assemblage of several biological components, such as plankton, epiphytes, macroalgae, fauna and sediment microbes. Therefore, the results in this study integrate the entire community as a way to undertake a more realistic approach.

**Fig 2:**
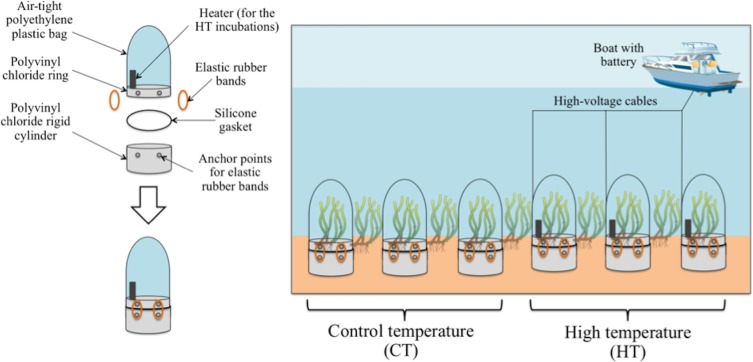
Simplified diagram of the incubations and of the experimental design. See detailed description in the text.

Incubations were similar to those used in previous studies analysing carbon metabolism and DOC fluxes *in situ* (e.g. [27, 55]), which consisted of two parts: a rigid cylinder made of a polyvinyl chloride (diameter = 20 cm; height = 17 cm) and an air–tight polyethylene plastic bag (height ≈ 37 cm; width ≈ 33 cm) attached to a polyvinyl chloride ring (width = 4 cm). Both parts are joined by a silicone gasket and tightly fastened by 4 elastic rubber bands (Fig 2). The rigid polyvinyl chloride cylinder was firmly inserted into the sediment (15 cm) though their sharpened lower end with only 2 cm of the cylinder above the sediment, which was the minimum necessary to fit the second part of the incubation over the upper end of the cylinder. The cylinders were inserted in the sediment between 1–2 hours before allocating the transparent plastic bag in the above part to reduce the effect of sediment perturbation. Each bag was provided with a sampling port located in the upper half of the bag (≈ 20 cm) to withdraw water samples. The walls of the bags (wall thickness ≈ 0.07 mm) were flexible enough to allow their movement with the hydrodynamics, preventing water stagnation. Light penetration measured inside the incubations was circa 99.15 ± 0.01% of incident light outside the bag. Oxygen diffusion controls were runs and demonstrated no oxygen permeability of the plastic bags. In addition, the three HT incubations had underwater heaters (Easyheater 25W; height ≈ 15.5 cm and width ≈ 4.5 cm) attached to the polyvinyl chloride rigid ring, separated from it 2.5 cm and at approximately 5 cm from the sea bottom to warm up circa 2° C the water during the experimental period (circa 24 hours). This value is within the range of sea surface temperature increase as a consequence of heat wave events (2–4°C; [12]). However, the experimentation time is lower than the minimum time of natural heat waves (2–3 days according to the World Meteorological Organization [11]). Longer experimental times may increase the chance of artefact occurrence associated with the use of incubation chambers (see the subheading *Limitations of the in situ methodology* in discussion section). Therefore, considered the sudden and temporary increase of temperature reached in this experiment as an approximation of the effect of natural heat waves on seagrass ecosystem should be done with caution.

To calculate the exact water volume in each incubation, 20 ml of a 0.1 M uranine solution (sodium fluorescein, C_20_H_10_Na_2_O_5_) was injected into each incubation bag at the end of the experiment, allowing 15 min for mixing, and shaking manually the bag to favour the quick mixing of the uranine. Thereafter, water samples were collected and kept frozen until spectrophotometric determination according to Morris et al. (2013) [56]. The mean volume of water enclosed in the incubations was 10.1 ± 0.5 l (*n* = 12). Incubations were placed in the evening just few hours before nightfall. To avoid the collection of resuspended material resulting from disturbance during installation of the experiment, the first sample was taken 2h after allocating the transparent incubation bag.

### Sampling procedure

To measure community carbon metabolism (through dissolved oxygen–DO–concentration) and DOC fluxes, water enclosed within each incubation was taken through the sampling port using a 50 ml acid–washed syringe (standard plastic previously subjected to blank control) at three times during the experiment: i) just before sunset (S1), ii) right after sunrise (S2) and iii) 6 h after sunrise (S3). In this way, community carbon metabolism and DOC fluxes in dark and light periods can be distinguished [27]. At the end of the experimental period, macrophyte biomass (i.e. the sum of seagrasses, epiphytes and macroalgae) inside the incubations were harvested, rinsed and dried to estimate the fresh community biomass (i.e. fresh weight; Kg FW m^-2^) in laboratory.

Along the experiment, temperature (°C) and light (lumens m^-2^) were continuously monitored with a HOBO data logger (UA-002-64) set in each incubation, and in bare sediment (n = 3) close to the experimental incubations. To transform lumens m^-2^ to μmol photons m^-2^ s^-1^, the most commonly conversion factor given in the literature under sunlight was used (1 lumens m^-2^ = 51.2 μmols photons m^-2^ s^-1^ [57]). Light daily dose was calculated using the average daily hours of light (photoperiod) in each station (14.33 and 10.65 h in summer and winter respectively). To better compare the two periods of study (winter and summer), sampling days in each season were chosen with similar tidal range as well as other environmental conditions (e.g. presence of clouds, no rain, wind, etc.) in order to reduce the environmental variability.

### Laboratory analysis

Water samples (15 ml) for DO concentration were fixed immediately after collection, kept in darkness, refrigerated and determined using a spectrophotometric modification of the Winkler titration method [58, 59]. Hourly rates of community respiration (CR^h^) were estimated as the difference in DO concentrations between samplings S2 and S1 divided by the time between both sampling using the following formula:

$${CR}^{h}\left(\frac{mmolO_{2}}{m^{2}\;d}\right)=\frac{{DO}_{S2}(\frac{mgO_{2}}{l})-{DO}_{S1}(\frac{mgO_{2}}{l})}{{\Delta T}_{T_{S1}-T_{S2}}(h)}*\frac{Vol(l)}{Area(m^{2})}*\frac{1}{32}\frac{mmolO_{2}}{mgO_{2}}$$

where DO_S2_ and DO_S1_ are the DO concentrations at sampling time S2 and S1, ΔT is the time elapsed between sampling times, “Vol” and “Area” are the measured volume and area of each benthic incubation respectively.

Hourly rates of net community production (NCP^h^) were estimated from the difference in DO concentrations between samplings S3 and S2 divided by the time between both sampling using the following formula:

$${NCP}^{h}\left(\frac{mmolO_{2}}{m^{2}\;d}\right)=\frac{{DO}_{S3}(\frac{mgO_{2}}{l})-{DO}_{S2}(\frac{mgO_{2}}{l})}{{\Delta T}_{S2-S3}(h)}*\frac{Vol(l)}{Area(m^{2})}*\frac{1}{32}\frac{mmolO_{2}}{mgO_{2}}$$

where DO_S3_ and DO_S2_ are the DO concentrations at sampling time S3 and S2, ΔT is the time elapsed between sampling times, “Vol” and “Area” are the measured volume and area of each benthic incubation respectively.

Hourly rates of gross primary production (GPP^h^) were computed as the sum of the hourly rates of CR and NCP (GPP^h^ = CR^h^ + NCP^h^). Finally, daily rates of gross primary production (GPP^d^), community respiration (CR^d^) and net community production (NCP^d^) were calculated using the following calculations (where photoperiod correspond to the number of sunlit hours in each sampling day):

$$GPP^{d}=GPP^{h}\;*\;Photoperiod\;\left(h\right);\;CR^{d}=CR^{h}\;*\;24h;\;NCP^{d}=GPP^{d}-CR^{d}$$

Metabolic rates in DO units were converted to carbon units assuming photosynthetic (PQ = moles O2:moles CO_2_) and respiratory quotients (RQ) of 1, values used widely in seagrasses studies (e.g. [36, 60, 61]).

DOC fluxes were estimated by changes in DOC concentration during light and dark periods. Water samples (20 ml) from benthic chambers were filtered through pre–combusted (450°C for 4 h) Whatman GF/F filters (0.7 μm) and were kept with 0.08 ml of H_3_PO_4_ (diluted 30%) at 4°C in acid–washed material (glass vials encapsulated with silicone–PTFE caps) until analyses. Concentrations of DOC were derived by catalytic oxidation at high temperature (720°C) and measured via NDIR detector using a Shimadzu TOC–VCPH analyzer. DOC–certified reference material (Low and Deep), provided by D. A. Hansell and W. Chen (University of Miami), of 41 to 45 of μmol DOC and 1 μmol DOC were used to assess the accuracy of the estimations (https://hansell-lab.rsmas.miami.edu/consensus-reference-material/index.html). The instrument blank ranged between 0 to 12 μmol DOC l^–1^ across the different analytical batches. Net DOC flux was calculated (according to Barrón & Duarte (2009) [27]; Egea et al. (2018) [28]) as the difference between the final and the initial DOC concentrations in the water samples. Then, the DOC flux was calculated using the following formula:

$$DOC\;flux\left(\frac{mmolC}{m^{2}\;h}\right)=\frac{DOC_{f}\left(\frac{mgC}{l}\right)-DOC_{0}\left(\frac{mgC}{l}\right)}{\Delta T_{T_{0}-T_{f}}\left(h\right)}*\frac{Vol\left(l\right)}{Area\left(m^{2}\right)}*\frac{1\;mmolC}{12\;mgC}$$

where DOC_f_ and DOC_0_ are the DOC concentrations at final (T_f_) and initial (T_0_) time, ΔT is the time elapsed between sampling times, “Vol” and “Area” are the measured volume and area of each incubation respectively.

Daily rates of DOC flux were calculated by the sum of the hourly DOC flux in light multiplied by photoperiod, and the hourly DOC flux in night multiplied by night hours. Thus, when net DOC flux was positive, the community was considered to act as a net DOC producer (i.e. source). When net DOC flux was negative, however, the community was considered to act as a net DOC consumer (i.e. sink).

### Data and statistical analysis

Prior to any statistical analysis, data were checked for normality (Shapiro–Wilk normality test) and homoscedasticity (Bartlett test of homogeneity of variance). When necessary, data were transformed to comply with these assumptions through natural logarithm. Even after several transformations, water temperature values did not meet the normality assumption; therefore, significant differences in water temperature among factors in each trial were analysed using the Kruskal–Wallis test with the Wilcoxon signed–rank test. The relationships between carbon community metabolism (GPP, CR and NCP) and temperature were analysed using the Spearman correlation. Statistical differences between factors (temperature and season) in carbon community metabolism (GPP, CR and NCP), *C*. *nodosa* biomass and DOC fluxes were analysed by a 2–way ANOVA test. When significant differences were found, a Tukey post–hoc test was applied to compare both the levels and interaction factors.

Data are presented as mean ± SE. The significance level (α) set in all tests performed was 0.05. Statistical analyses were computed with R statistical software 3.0.2 (R Development Core Team 2013).

## Results

### Abiotic parameters and benthic communities

Mean water temperature in CT treatment varied between 16.5 ± 0.02°C in winter to 24.6 ± 0.03°C in summer. The heaters create a constant offset from the ambient water temperature and then, the HT treatments had a day–night temperature oscillation. Water in HT treatment was statistically higher in both sampling events (circa 2°C; *p* < 0.001) when compared to CT treatment, averaging 18.4 ± 0.02°C and 26.7 ± 0.03°C in winter and summer respectively (Table 1). Underwater daily irradiance at the canopy level of *C*. *nodosa* meadow at midday was 147 ± 13 μmol photons m^–2^ s^–1^ during the winter trial and 260 ± 18 μmol photons m^–2^ s^–1^ during the summer trial. The average community biomass (i.e. the sum of seagrasses, epiphytes and macroalgae) was similar between treatments in both season and significantly higher (*p* < 0.004) in the summer trial (1.5 ± 0.2 Kg FW m^-2^) than in the winter one (1.0 ± 0.1 Kg FW m^-2^) (Table 1). *Cymodocea nodosa* was clearly the dominant macrophyte in the meadow, since the biomass of macroalgae and epiphytes in both treatments and seasons were negligible.

**Table 1:** Community biomass (Kg fresh weight m^-2^) and water temperature (°C) in the different treatments and seasons.

Season	Treatment	Community biomass (Kg FW m^-2^)	Water temperature (°C)	Temperature range (°C)	Temperature increase (°C)
Winter	CT	0.92 ± 0.07^a^	16.5 ± 0.02^a^	16–17.4	-
	HT	1.03 ± 0.05^a^	18.4 ± 0.03^b^	17.9–18.9	1.9 ± 0.01
Summer	CT	1.78 ± 0.24^b^	24.6 ± 0.02^c^	23.9–25.3	-
	HT	1.23 ± 0.09^b^	26.7 ± 0.03^d^	26–27.3	2.1 ± 0.01

All data are expressed as mean ± SE. CT: Control temperature; HT: High temperature. Superscript letters (a, b, c, d) indicate significant differences between treatments and seasons at α = 0.05.

### Effects on community metabolism

**Fig 3:**
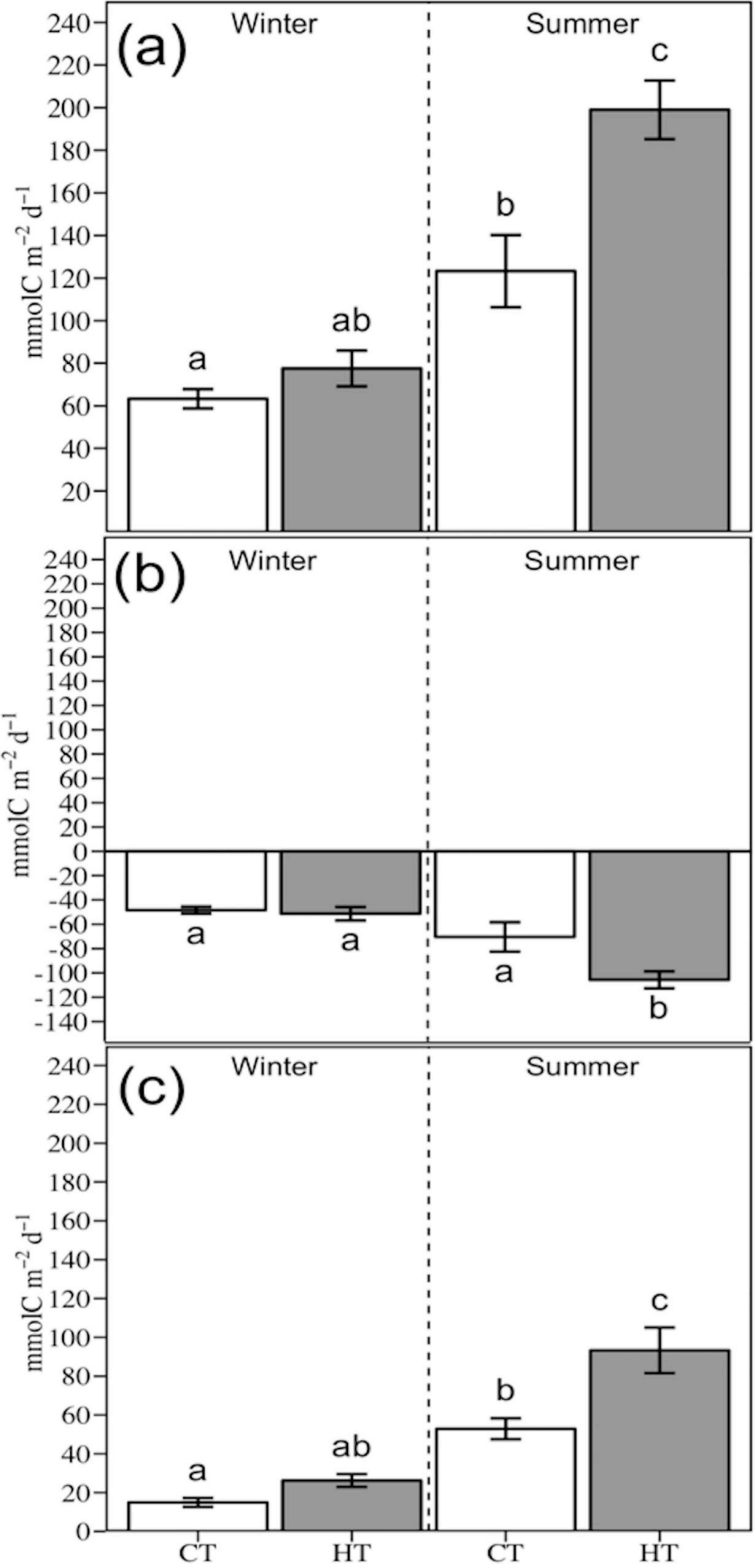
**Effect of sudden and temporary increase in temperature on (a) Gross Primary Production (GPP), (b) Community Respiration (CR) and (c) Net Community Production (NCP) in winter and summer.** CT: Control temperature; HT: High temperature. Different letters indicate significant differences between treatments and seasons. Data are expressed as mean ± SE (*n* = 3).

**Fig 4:**
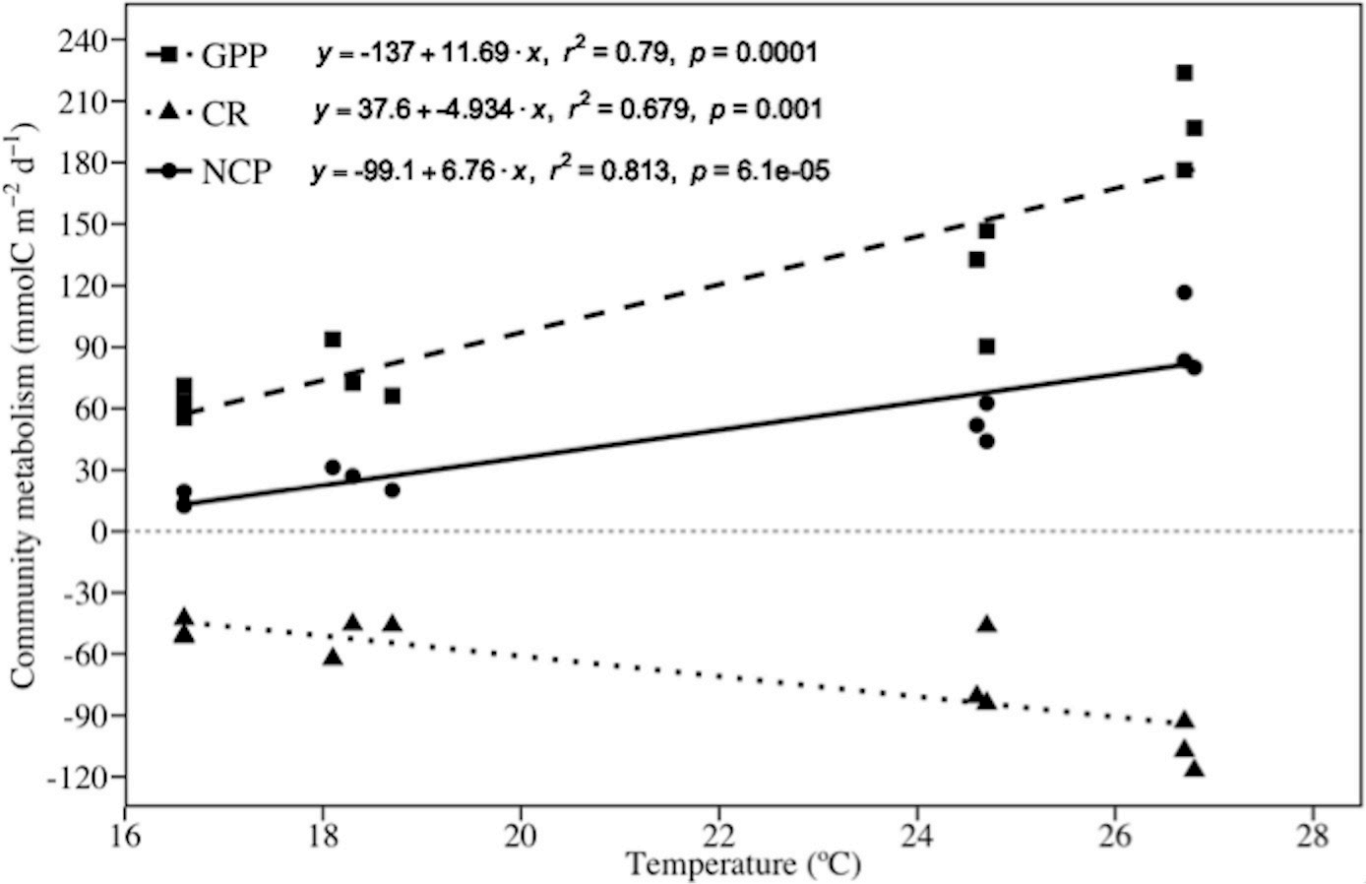
Relationship between carbon community metabolism and temperature on Gross Primary Production (GPP), Community Respiration (CR) and Net Community Production (NCP).

High temperature treatments produced an increase in the Productivity:Respiration (P:R) ratio of 15% during the winter trial (from 1.3 ± 0.04 to 1.5 ± 0.05) and 6% in the summer trial (from 1.8 ± 0.09 to 1.9 ± 0.12). The GPP and NCP in CT were significantly higher in summer than in the winter trial. Temperature increase only affected significantly to the GPP, CR and NCP during the summer but not in the winter trial. Hence, GPP, CR and NCP were ca. 1.6, 1.5, 1.8 times higher under HT than under CT in the summer trial (Fig 3 and Table 2). Overall, when using all the temperature data (i.e. winter, summer, CT and HT) a linear correlation between carbon community metabolism (i.e. GPP, CR and NCP) with temperature was found along the experimental period (Table 3 and Fig 4).

**Table 2:** Results of the 2–way ANOVA analysis of the factors temperature and season in the carbon community metabolism.

GPP				
	df	MS	F	*p*
Season	1	24703	58	**<0.001**
Temperature	1	6072	14	**0.005**
Season & Temperature	1	2839	6.687	**0.032**
Residuals	8	425		
CR				
	df	MS	F	*p*
Season	1	4380	25	**0.001**
Temperature	1	1099	6	**0.037**
Season & Temperature	1	785	4.485	0.067
Residuals	8	175		
NCP				
	df	MS	F	*p*
Season	1	8279	61	**<0.001**
Temperature	1	2004	15	**0.005**
Season & Temperature	1	638	4.669	0.063
Residuals	8	137		

GPP: gross primary production; CR: community respiration; NCP: net community production. Bold numbers indicate significant differences at α = 0.05.

**Table 3:** Spearman correlations between carbon community metabolism (mmolC m^-2^ d^-1^) and temperature (°C) along the experimental period.

GPP		CR		NCP	
r	*p*	r	*p*	r	*p*
0.88	0.0004*	-0.74	0.0065*	0.93	0.0001*

GPP: Gross primary production; CR: Community Respiration; NCP: Net community production. *r* is the correlation coefficient. Asterisks (*) indicate significant differences at *p* < 0.05.

### DOC fluxes

**Fig 5:**
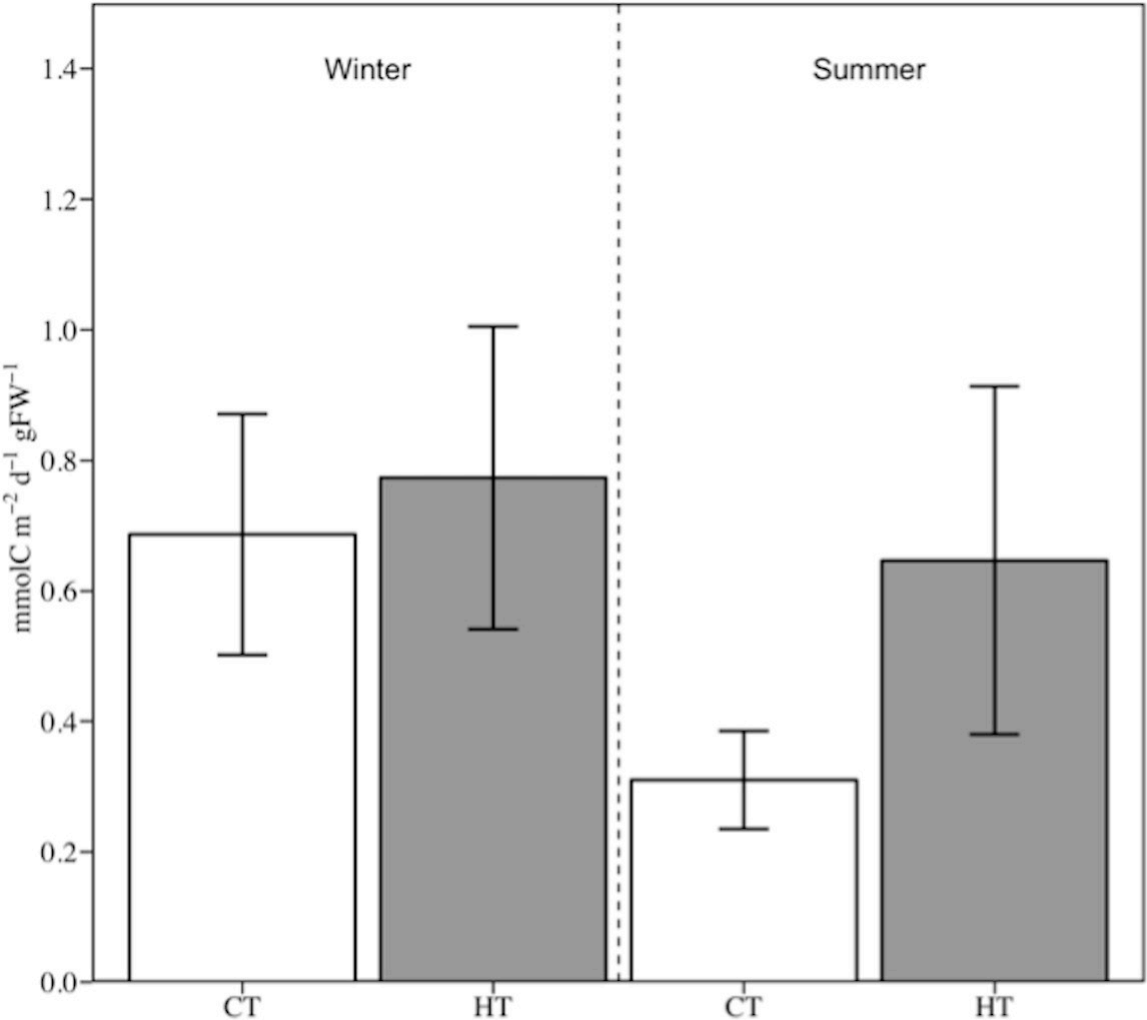
Effect of sudden and temporary increase in temperature on net DOC fluxes in winter and summer. CT: Control temperature; HT: High temperature. Data are expressed as mean ± SE (*n* = 3).

The dissolved organic carbon flux was similar in CT and HT treatments in the winter trial. In contrast, during the summer trial, the DOC flux in HT doubled that of the CT, although differences were not statistically significant (Fig 5 and Table 4). Overall, DOC fluxes ranged from ca. 25–30% of NCP in the summer trial to ca. 100% of NCP in the winter trial and even exceeding the NCP under CT (126% of NCP).

**Table 4:** Results of the 2–way ANOVA analysis of the factors temperature and season in the dissolved organic carbon (DOC) fluxes.

	df	MS	F	*p*
Season	1	0.19027	1.538	0.250
Temperature	1	0.13548	1.095	0.326
Season & Temperature	1	0.04746	0.384	0.553
Residuals	8	0.12370		

## Discussion

This study showed that a sudden and temporary increase in water temperature *in situ* had significant consequences in the carbon dynamic of seagrass communities, especially during the summer. During this season, simulated temperature increased significantly the carbon community metabolism (Fig 3 and Table 2), meanwhile DOC fluxes was twice of the control although without significant statistical differences (Fig 5). In contrast, there were no significant differences in the carbon community metabolism and DOC fluxes mediated by temperature increase during the winter trial.

### Community carbon metabolism

The community dominated by *Cymodocea nodosa* was highly autotrophic in both treatments (control and high temperature) and seasons (winter and summer), which is in agreement with previous findings in the same location [32], and within the range of values described by Duarte et al. (2010) [36]. Our results showed that a sudden and temporary increase in temperature does not only affect to the seagrass *C*. *nodosa* but also to the whole community (e.g. plankton, epiphytes, macroalgae, fauna and sediment microorganisms). The net community production significantly increased under high temperature when compared to the CT treatment in summer (Fig 3C and Table 2). Moreover, we found a positive correlation between temperature and seagrass production (Fig 4). Then, the effect of temperature on photosynthetic rate was positive, which triggered a higher increase in GPP when compared to CR (Fig 3A & 3B). This result is according with the pattern found in some terrestrial plants researches (as noted in the Davidson & Janssens (2006) review [62]) and in previous studies in seagrasses such as Adams et al. (2017) [56]. This result evidences that a higher frequency of short–term increase of temperature (few days) in the next decades may produce an increase in the seagrass community production when they are not close to their thermal tolerance limit, or when they are not subjected to another stressor (e.g. light limitation, eutrophication, etc.), as the occurrence of additive or non–additive (i.e. synergistic or antagonistic) responses to multiple stressors can be produced in the system [41, 63–65]. An increase in the net community production can trigger noteworthy consequences to the whole seagrass community, as this becomes the community more autotrophic. This carbon uptake surplus may help seagrasses to synthesize more carbon skeletons that can be directly used for growth or stored, supporting seagrass growth during unfavourable growing conditions [66–68]. However, it must be considered that our results were achieved in a healthy community where *C*. *nodosa* evidences high density and biomass and inhabits sandy/muddy sediments with medium-low organic matter content (2%) [69–72]. In other communities where the ratio macrophyte biomass *vs* sediment was lower or the organic matter content in sediments higher, the effect of temperature over respiratory processes will probably gain more relevance [32, 73].

In contrast to summer, there were no significant differences in the carbon community metabolism and DOC fluxes between treatments during the winter trial. The *C*. *nodosa* population may be far from its optimum temperature for growth during the winter but very close during the summer [74], and therefore opposite trends can be expected, as it was evidenced by our findings (i.e. an improvement of net community production and DOC release in winter). Apart from water temperature and seagrass biomass, one of the major differences between both seasons is the light doses received at the top of the canopy, being ca. 240% higher in summer than in winter (ca. 13.3 ± 0.9 mol photons m^–2^ d^–1^ in summer and ca. 5.6 ± 0.5 mol photons m^–2^ d^–1^ in winter). Thus, the limited response of *C*. *nodosa* community to temperature increase recorded in winter may be due to the remarkable lower temperature, biomass and light incidence occurring in this season, which prevents photosynthetic organisms in the community from increasing their metabolism under higher temperature levels, as light limitation is one of the most important factors for the metabolism of seagrass communities [75–79], and has also been recently suggested as a key factor in the DOC release by coastal marine communities [35].

### Community dissolved organic carbon (DOC) fluxes

In the present study, winter and summer DOC fluxes in the control temperature treatment were ca. 310 and 680 μmol C gFW^-1^ m^-2^ d^-1^, which is similar to the net DOC fluxes recorded in both seasons in an annual study for this species in the same location [32], and within the range of values reported by Barrón et al. (2014) [35]. These results represented ca. 30% and 126% of the measured NCP respectively. Previous studies have shown that the net DOC flux in seagrass communities was significantly correlated with water temperature [27, 35], but it is important to note that these studies are based on seasonal monitoring programs. Barrón et al. (2014) [35] indicated that each degree of temperature increase led to an increase of about 1.5 mmol C m^−2^ d^−1^ in the net DOC flux in *Posidonia oceanica* stands. The results of the present study confirmed this relationship between temperature and the DOC released in seagrass communities, which was independent of the season. Thus, an increase of ca. 3 mmol C m^−2^ d^−1^ by each degree of temperature raised was recorded here for *C*. *nodosa*, both in winter and in summer. However, as a consequence of the significant increase in seagrass biomass in the summer trial compared with the winter one, which is in line with previous studies (e.g. [76, 80]), the results vary when normalized by the macrophyte biomass. In this case, we still recorded a weak increase (13%) in DOC release in winter (from ca. 680 to 770 μmol C gFW^-1^ m^-2^ d^-1^) in contrast to the summer when the net DOC flux doubled (from ca. 310 to ca. 650 μmol C gFW^-1^ m^-2^ d^-1^) although no significant statistical differences were found.

The absence of significant statistical differences in DOC fluxes was mainly attributed to the large variability between replicates. DOC measurements are very sensitive to biases derived from the manipulation of the community (e.g. remobilization of sediment and rupture of rhizomes when incubation was placed) and sampling (e.g. possible contamination when samples are fixed and transported; 27), which could be avoided increasing the number of independent replicates in our experimental design and, thus contributing to increase the robustness of our conclusions. However, the complexity of the experimental setup and sampling procedure made us to restrict the number of replicates. In addition, the experimental time was low (less than 24 h), which can also limit the response of the community, but as explained below (see the subheading *Limitations of the in situ methodology*), the use of the incubation chambers may produce large artefacts if the cultivation time increases. In spite of this lack of statistical differences, short–term temperature increase prompted large differences in DOC fluxes, which highlights the direct effect of temperature in DOC fluxes. This can have important ecological implications, since larger DOC release from seagrass stands means a quicker and more efficient transference of carbon and energy from primary producers to higher trophic levels (e.g. plankton community) [27, 32, 33], which may boost secondary production under such conditions.

### Temporary increase of temperature *in situ* as an approximation of the effect of marine heat waves

The present work analysed for the first time the effects of a sudden and temporary increase of temperature *in situ*–which characterizes the marine heat waves–in a key and threatened coastal habitat such as seagrass communities. We acknowledged that although the temperature increase in this experiment (circa 2° C) is within the range of sea surface temperature increase during heat wave events (2–4°C; [12]), the experimentation period is lower than the minimum time estimated in natural heat waves (2–3 days according to the World Meteorological Organization [11]). However, longer experimental periods may increase the chance of artefact occurrence associated with the use of incubation chambers (see the subheading *Limitations of the in situ methodology* in discussion section), making difficult to increase temperature *in situ* for a longer period. However, it is important to note that despite the limited exposure time to enhanced temperatures, significant differences and important trends have been recorded both in the carbon metabolism and in the DOC fluxes of seagrass communities. Real marine heat waves lasting more than 24 hours probably shall trigger greater differences than that found in this experiment. The consideration of the sudden and temporary increase of temperature reached in this experiment as an heat wave should be done with caution. However, we consider that the results obtained in this work using this pioneering methodology provided some interesting and novel results regarding the likely community–level effects of a sudden and temporary temperature increment.

Most of the previous studies regarding marine heat waves in seagrasses showed negative consequences, including shoot mortality and dieback [22, 52, 53, 81], results not found in the present work. These previous studies were usually based on data collected after marine heat wave events or marine heat waves stress induced experimentally under mesocosm conditions. In the first case, the results reported are probably a consequence of the interaction between elevated temperatures and other factors such as light limitation, as noted by different authors [52, 53], or because heat waves affected to species living at the extreme of their thermal tolerance region [53]. In the second case, studied usually focused on the ecophysiological response of isolated seagrass plants, without considering the whole community and their interactions, buffer capacities and feedbacks, and the results may be subjected to uncertainties derived from seagrass manipulation and possible artefacts associated with the experimental design. In contrast, the present work makes an *in situ* approximation at the community level, with a minimum disturbance of natural seagrass communities, and hence can be considered as a more integrative response and closer to the natural conditions, although it is not exempt from methodological limitations.

Our results showed that community responses to heat waves may be not as harmful as previously believed, which is in line with some previous studies in terrestrial grassland communities (e.g. [82, 83]). These studies demonstrates that community responses to heat waves are not necessarily negative, and plant conditions can even improve under certain conditions (e.g. increase the leaf relative growth, leaf chlorophyll content and plant development; [45, 84]). In most of the studies the negative effects of heat waves were linked to the combination with other stressors (e.g. drought; [82]), the successive frequency of heat wave events [84], season [45, 83] or depended on the plant species [83] and plant cover [85]. The results of our work indicate that a sudden and temporary increase in temperature may enhanced the productivity and DOC fluxes in seagrass communities. Thus, the effects of this sudden and temporary increase in temperature (as a proxy of heat waves) in seagrasses can be heterogeneous (positive, negative or neutral) depending on the dominant seagrass species, period and in the interaction with other stressors. Therefore, the effects of heat waves in seagrasses should deserve future studies using *in situ* experimental approaches in different seagrass species, periods and locations, in order to gain more knowledge in how this valuable ecosystem will respond to the increase of this kind of extreme climatic event.

### Limitations of the *in situ* incubation methodology

The methodology used in this experiment has been widely utilized (e.g. [27, 55]), because it allows an effective approach of the *in situ* metabolic responses of the whole community. This methodology, however, has implicit some limitations which may underestimate or overestimate the net community production (NCP) in the incubations as a consequence of the isolation of the community inside. Thus, pH and dissolved oxygen may increase as a result of the photosynthetic activity inside the long–term incubations, which do not occur in the same degree in natural meadows where the turbulent mixing avoids the oversaturation [86]. This may enhance carbon limitation and favour photorespiration further decreasing the photosynthetic rates and yielding an underestimation of NCP [87]. Nonetheless, a recent study in *P*. *oceanica* underscored that these uncertainties usually are produced in experiments where NCP is estimated at solar noon or during several hours (more than six) [88]. In our experimental set–up, the NCP was estimated during 6 hours after sunrise, which can underestimate up to 25% the NCP (i.e. according to Olivé et al. (2016) [88]). We also assumed respiratory quotients of 1 (RQ = 1). In shallow estuaries, RQ can be higher than 1 triggering an underestimation in NCP, and especially under anaerobic conditions, where RQ values usually range between 1.0 to 2.0 [89]. However as no anaerobic conditions occurred during the experiment (S1 Table), and the reported range for respiratory quotients in seagrass communities is between 0.8–1.14 [90], we have adopted an RQ of 1 for simplicity and consistency with previous studies [36, 60, 61]. On the other hand, the methodology used in this work may also overestimate the NCP of the communities. For example, community respiration can be underestimated under low oxygen conditions, in dark incubation during long periods of time [91, 92]. However, the average DO concentrations measured in S2 period were higher than the accepted 2 mg O_2_ l^−1^ threshold for hypoxia [93] in all treatments and seasons (S1 Table). In summary, it is possible that the NCP estimated in this study may have a certain degree of underestimation as a result of the isolation of the community inside the incubation, which indicates that this community may be even more autotrophic than suggested by our results.

The complexity of this experimental design *in situ* resulted in a low (i.e., three) number of independent replicates for each treatment, although enough to evaluate statistically the ecological response to a disturbance. However, more replicates or the replication of the experiment in other areas in the bay would have enhanced the robustness of our results. Part of the non–significant records found here, especially regarding DOC release, may be a consequence of this reduced number of replicas used. Manipulating water temperatures *in situ* is a logistic challenge, which has not yet been addressed probably as a consequence of the technical difficulties and costs required to induce an increase of temperature in the sea. However, this is an important step for understanding how natural communities respond to thermal stress. Previous studies in seagrass meadows focusing on thermal stress *in situ*, were developed close to thermal effluents from power stations [41, 94–97]. However, using this approach does not guarantee that temperature was the only manipulated factor, since physicochemical characteristics of the effluents can be also altered (e.g. salinity, turbidity, hydrodynamic, presence of pollutants, etc.), and real replication is difficult to reach because power stations usually have only one effluent. Therefore, although our approach has some technical limitations, at least allowed for real independent replication and for the modification of a single factor (i.e. temperature).

## Conclusions

This research evidenced that those communities dominated by seagrasses are very sensitive to sudden and temporary increase of temperature. Our results showed that an eventual and short–term increase of temperature may be not as harmful as previously believed, and may even increase the community production and DOC release. Although taking into account the limitations aforementioned, our study can be used as a proxy of the effects of marine heat wave events, since we used a pioneering methodology to simulate *in situ* a sudden and temporary increase of temperature in the whole community. Thus, this study indicates that short-term marine heat wave events in temperate areas may make more autotrophic the carbon metabolism of seagrass communities and can yield an increase in the DOC released. However, this finding has to be restricted to this temperate seagrass community, which do not live close to their thermal tolerance limit, and therefore further research following this integrative *in situ* approach should be done in communities bearing different species and from different bioregions.

## Supporting information

S1 Table Raw dissolved oxygen values (DO; mgO_2_ l^-1^) recorded in the incubations chambers in control temperature (CT) and high temperature (HT) treatments at three times during the day: i) just before sunset (S1), ii) right after sunrise (S2) and iii) 6 h after sunrise (S3). (DOCX)
