# Supplementary material for: Effect of In Situ short–term temperature increase on carbon metabolism and dissolved organic carbon (DOC) fluxes in a community dominated by the seagrass Cymodocea nodosa
Source: PLoS One. 2019 Jan 14;14(1):e0210386. doi: 10.1371/journal.pone.0210386 (PMC6331083; doi:10.1371/journal.pone.0210386)
Supplement: S1 Table — (DOCX) [file pone.0210386.s001.docx]

|  |  | DO (mg l^-1^) | | |
| --- | --- | --- | --- | --- |
| Season | Treatment | S1 | S2 | S3 |
| Winter | CT | 14.3 ± 0.3 | 10.4 ± 0.4 | 12.3 ± 0.4 |
|  | HT | 13.7 ± 0.1 | 9.5 ± 0.4 | 12.1 ± 0.1 |
| Summer | CT | 9.9 ± 0.1 | 6.5 ± 0.6 | 9.5 ± 0.3 |
|  | HT | 9.8 ± 0.1 | 4.8 ± 0.3 | 9.5 ± 0.4 |
